# Cationic Curcumin Nanocrystals Liposomes for Improved Oral Bioavailability: Formulation Development, Optimization, In Vitro and In Vivo Evaluation

**DOI:** 10.3390/pharmaceutics16091155

**Published:** 2024-08-31

**Authors:** Xiang Cheng, Xiaoran Han, Jia Si, Cong Dong, Zhongjuan Ji, Shicong Zhao, Xiangting Wu, Haiyan Li, Xiangqun Jin

**Affiliations:** 1 Department of Pharmacy, Jilin University, Changchun 130021, China; chengxiang22@mails.jlu.edu.cn (X.C.); hanxr22@mails.jlu.edu.cn (X.H.); jiasi23@mails.jlu.edu.cn (J.S.); jizj23@mails.jlu.edu.cn (Z.J.); dongcong23@mails.jlu.edu.cn (C.D.); sczhao@jlu.edu.cn (S.Z.); xiangting21@mails.jlu.edu.cn (X.W.); 2 Department of Pharmacy, Changchun University of Chinese Medicine, Changchun 130021, China; hyl09@mails.jlu.edu.cn

**Keywords:** curcumin, cationic nanocrystals, oral absorption, bioavailability enhancement, mucous barrier

## Abstract

Curcumin, a naturally occurring poorly water-soluble polyphenol with a broad spectrum, is a typical BCS IV drug. The objective of this study was to develop curcumin nanocrystals liposomes with the aim of improving bioavailability. In this study, we prepared cationic curcumin nanocrystals with a particle size of only 29.42 nm; such a phenomenal range of particle sizes is very rare. Moreover, we summarized and evaluated the parameters of the nanocrystal preparation process, including methods, formulations, etc., and the rules we concluded can be generalized to other nanocrystal preparation processes. To counteract the instability of the nanocrystals in the digestive tract, cationic curcumin nanocrystals were loaded into negatively charged liposomes through gravitational force between different charges. Unexpectedly, chitosan oligosaccharide was found to promote the self-assembly process of curcumin nanocrystal liposomes. In vitro and in vivo experiments demonstrated that chitosan-modified curcumin nanocrystal liposomes exhibited enhanced resistance to enzyme barriers, mucus barriers, and cellular barriers, resulting in a 5.4-fold increase in bioavailability compared to crude powder formulations. It can be concluded that cationic nanocrystals liposomes represent an appropriate novel strategy for improving the dissolution rate and bioavailability of poorly soluble natural products such as curcumin.

## 1. Introduction

Curcumin (CUR), a primary constituent of turmeric, is commonly used as a spice in Indian cuisine [1]. Moreover, it finds widespread application in the food, medical, and healthcare industries. In recent decades, burgeoning interest in curcumin has led to extensive research, consistently showcasing its beneficial anti-inflammatory, antibacterial, hepatoprotective, antioxidant, and wound-healing properties [2,3,4]. Additionally, a growing body of clinical trials has affirmed CUR’s efficacy in combatting various cancers, such as lung and colon cancer [5,6]. However, the low solubility, limited bioavailability, and instability of CUR’s conjugated polyene structure in water pose substantial challenges to its utilization [7]. Hence, exploring its full potential through nanoformulation development is imperative.

Recent research in nanomedicine has concentrated on the design of smaller and more-stable particles and the optimization of their surface properties with a view to enhancing the therapeutic effects of drugs. Nonetheless, while most nanomedicines enhance drug bioavailability, their practical application is often hindered by their low drug-carrying capacity [8], typically less than 10%, necessitating significant carrier material usage to maintain the drug’s nanostate, potentially leading to adverse effects. In contrast, drugs based on nanocrystals, with their nearly 100% drug loading capacity and robust plasticity, offer efficient therapeutic outcomes with only a minimal number of particles [9]. Consequently, an increasing number of drugs are being considered for delivery in the form of nanocrystals (NCs), including CUR, bioflavonoids [10], pomalidomide [11], paclitaxel [12,13], and berberine [14].

The techniques for producing nanosized crystalline particles can be categorized into top-down methods, such as milling and high-pressure homogenization, and bottom-up approaches like precipitation and self-assembly [15]. Each preparation method has its inherent advantages and disadvantages. For instance, high-pressure homogenization is universally applicable, enabling particle size reduction by adjusting the number of homogenizations and pressure [16]. Conversely, precipitation, while simple and cost-effective, necessitates the use of numerous solvents unavoidably. Moreover, a significant challenge with nanocrystalline drugs is poor stability, characterized by crystal aggregation, growth, and precipitation, which must be addressed. The most typical method by of achieving stable NCs involves adding stabilizers to the nanosuspension, preventing aggregation and sedimentation by enhancing the surface charge and electrostatic repulsion between the crystals. However, different stabilizers yield crystals with distinct shapes, surface charges, particle sizes, and stability—crucial factors in product evaluation.

Nanocrystals are formed by the stacking of π–π bonds between drug molecules and the interactions of van der Waals forces, hydrogen bonding, and other similar forces [12,17]. This state can only exist in a specific system in a relatively stable equilibrium. However, as soon as the nanocrystals enter the gastrointestinal tract, the blood, the abdominal cavity, and other environments, this equilibrium is broken. For example, Na^+^ and Cl^−^ will break Van der Waals forces between molecules, which results in the nanocrystals aggregating into clusters in a short period of time and precipitating, thus losing the nano-properties of the drug. Although this method of drug delivery can still improve the oral bioavailability of BCS II and BCS IV drugs compared to the original powder, it is far from the desired effect in practice. Significantly, BCS II and BCS IV drugs encounter distinct challenges. Mere enhancement of drug dissolution does not address the issue of low bioavailability in BCS IV medications, attributed to P-glycoprotein (P-gp) exocytosis [18]. This necessitates the absorption of nanocrystals as complete entities to prevent premature dissolution during absorption. Furthermore, when nanocrystalline drugs are injected into the blood circulation, the clustered crystals will block the capillaries of the body, leading to end-circulation obstacles. Consequently, the initial challenge is to enhance the stability of nanocrystalline drugs. Xu, Z et al. demonstrated the stabilizing effect of rhodopsin nanocrystals by incorporating them into hydrogels [19]. Wang, Y et al. achieved a significant improvement in the oral bioavailability of etoposide by utilizing liposome-loaded etoposide nanocrystals [20]. Moreover, research suggests that cationic nanoparticles exhibit enhanced absorption by Caco-2 cells upon oral administration compared to anionic particles [21]. It is therefore anticipated that the development of cationic nanocrystalline liposomes will facilitate the delivery of orally administered drugs, overcoming the three major barriers that such drugs must overcome: (1) enzyme barrier; (2) mucus barrier; (3) cellular barrier.

In this study, we optimized the preparation process of CUR-NCs to produce cationic CUR-NCs with a size of 29.42 nm, while CUR-NCs prepared by peers are generally around 200 nm. Throughout NCs preparation, we delineated the general principles governing the influence of environmental and formulation factors on crystal size, conducting a series of investigations, including TEM, PXRD, and stability analyses, on the prepared NCs. In older to improve the stability of curcumin nanocrystals, we loaded cationic nanocrystals into anionic liposomes via the mutual attraction between different charges. Pharmacokinetic assessments, in vitro dissolution tests, and particle mucus diffusion experiments confirmed the superior absorption of cationic curcumin nanocrystals (CUR-NCs^+^) compared to curcumin coarse powders (CUR-CCs), particularly highlighting the enhanced gastrointestinal mucosal penetration and oral absorption efficiency of CUR-NCs^+^ over their anionic counterparts. Furthermore, following liposomal encapsulation, the capacity of CUR-NCs^+^ to penetrate all three barriers was markedly enhanced, with a 5.4-fold increase in bioavailability compared to CUR-CCs.

## 2. Materials and Methods

### 2.1. Materials

CUR, domiphen bromide (DPB), NaOH, mannitol, lecithin from egg yolk pure, vitamin E-TPGS, pepsin, trypsin, coumarin 6, ethanol, chitosan oligosaccharide (Macklin Chemical Technology Co., Ltd., Shanghai, China); hydroxypropyl cellulose (HPMC), Sodium dodecyl sulphate (SDS), polyethylene glycol (PEG-1000), Plasdone K30 (PVP K30), sodium tylose (CMC-Na) (Tianjin Guangfu Fine Chemical Research Institute, Tianjin, China); polyvinyl alcohol (PVA), Benzalkonium Chloride (BZC) (Jiangxi Alpha Hi-tech Pharmaceutical Co., Ltd., Wuxi, China); hexadecyl trimethyl ammonium bromide (CTAB) (Beijing Mreda Technology Co., Ltd., Beijing, China); dimethyl sulfoxide (DMSO), methanol, acetone, and acetonitrile of chromatographic grade (BSZH Scientific Inc, Beijing, China). All reagents and chemicals used were of analytical or chromatographic grade. HT29-MTX-E12 (E12) cells, Caco-2 cell (Shanghai EK-Bioscience Biotechnology Co., Ltd., Shanghai, China). DMEM, DiI, DiO, Mucin from porcine stomach (Dalian Meilun Biotechnology Co., Ltd., Dalian, China).

### 2.2. Analysis Method

CUR was analyzed via HPLC. Chromatographic separation was achieved on Acchrom Tech s3000 with an Inert Sustain Swift C18 (250 mm, 4.6 mm, 5 μm) column using acetonitrile–4% acetic acid water 48:52 (*v*/*v*) as a mobile phase with a flow rate of 1 mL/min, detected at 430 nm [22].

### 2.3. Fabrication of Nanocrystals

This study assessed five common techniques for preparing NCs: milling (MIL); high-speed shearing (HSS); sonoprecipitation (SOP); freeze-drying (FRD); and sonoprecipitation combined with high-pressure homogenization (HPH).

In summary, HSS involves adding CUR-CCs to a high-speed shearing machine to yield smaller NCs. FRD entails dissolving CUR in DMSO, adding it to an aqueous solution with surfactant and freeze-drying protectant, rapidly solidifying it with liquid nitrogen, and lyophilizing to obtain the sample. HPH involves adding CUR-NCs (prepared via SOP) into a high-pressure homogenizer and homogenizing several times to achieve smaller NCs. SOP consists of dissolving CUR in DMSO, adding the solution to water with a surfactant under low-temperature ultrasound, centrifuging to remove the supernatant, and resuspending in water to obtain CUR-NCs.

This study optimized the preparation process of CUR-NCs by varying nearly ten parameters, using size, polydispersity index (PDI), and zeta potential as evaluation indicators.

Initially, different organic phases, including DMSO, acetone, ethanol, and 0.5 M NaOH, were tested by dissolving 0.2 g CUR in 20 mL of each, adding it in a 1:10 ratio to 0.25% PVP K30 solution at room temperature, and sonicating for 15 min to obtain the sample.

Subsequently, using the same parameters, nine surfactants were screened with DMSO as the organic phase to assess their impact on sample size and PDI.

Additionally, this study investigated the effects of temperature, ultrasonic time, phase ratio, and surfactant concentration on CUR-NCs size. The specific parameters are detailed in Table 1.

Furthermore, the effects of drug concentration, scaling factor, sample preparation vessel, and centrifugal resuspension on CUR-NCs size and PDI were explored.

### 2.4. Fabrication of Liposome

The CUR-NCs@Lipo were prepared using the thin film hydration method [23]. Specifically, 300 mg of lecithin, 60 mg of cholesterol, and 40 mg of vitamin E-TPGS were dissolved in 40 mL of chloroform. The chloroform was removed via rotary evaporation, and the centrifugally resuspended CUR-NCs^+^ were added. CUR-NCs@Lipo were then prepared by incubating the mixture at 4 °C on a shaker for 2 h, vortexing for 3 min, sonicating for 5 min, and filtering it through a 220 nm membrane. Subsequently, the particle size, PDI, and surface potential of CUR-NCs@Lipo were measured. Successful encapsulation was confirmed by observing a change in charge. CUR-NCs@Lipo were further combined with a 1% chitosan oligosaccharide solution in a 1:1 ratio to form CUR-NCs@Lipo-CS.

### 2.5. Characterization

#### 2.5.1. Size, Zeta, and PDI

Size, zeta, and PDI were determined using a Malvern Nano-ZS instrument (Malvern Panalytical Ltd., Enigma Business Park, UK). Before each measurement, the samples underwent dilution with distilled water to achieve an optimal scattering intensity, followed by manual redispersion through shaking [24,25]. Triplicate samples were analyzed for size, PDI, and zeta potential, with each test conducted over ten repetitions at 4 °C.

#### 2.5.2. Powder X-ray Diffraction (PXRD)

Add 5% lyoprotectant (mannitol) to the CUR-NCs, pre-freeze at −20 °C for 24 h, then freeze-dry for 60 h. The PXRD patterns of the CUR nanopowder were analyzed using Oxford Diffraction equipment (Oxford Diffraction Ltd., Abingdon, UK). Data were collected with Cu-Kα radiation at 40 kV voltage, with a scanning speed of 1°/min over a range from 5° to 90° [26].

#### 2.5.3. Transmission Electron Microscope (TEM)

Position the copper grid, covered with a carbon support film, face up on a clean sealing film. Using a pipette, apply 5 μL of 0.75 mg/mL CUR-NCs^+^, CUR-NCs^−^, CUR-NCs@Lipo, and CUR-NCs@Lipo-CS onto the copper grid; allow it to stand for 10 min, then remove excess liquid with filter paper. Absorb any remaining liquid from the copper mesh. Next, use a pipette to apply 5 μL of 3% phosphotungstic acid dye (pH 7.0) onto the sealing film. Invert the copper mesh onto the phosphotungstic acid droplets and let it rest for 1 min. Absorb any excess liquid from the copper mesh with filter paper. Air-dry the grid at room temperature for 10 min before placing it in a transmission electron microscope (HT7800) for imaging and analysis [27].

Import the TEM images into ImageJ (2.14.0) software for analysis. Measure the size of no fewer than 200 particles, analyze the data obtained, and represent the size of CUR-NCs using the mean value (*Z_D_*) [28]. Calculate the *PDI* of CUR-NCs according to Equation (1):

(1) $$PDI={\left(\frac{Sigma}{Z_{D}}\right)}^{2},$$

where *sigma* is the standard deviation.

#### 2.5.4. Saturated Solubility

In brief, excess CUR-CCs, CUR-NCs^+^, and CUR-NCs^−^ were added to distilled water, vortexed for 5 min, and agitated at 37 °C for 12 h on a shaking table. The resulting mixture was centrifuged at 14,000 rpm for 12 min, the supernatant was collected, filtered through a 220 nm microporous filter, and the concentration was analyzed via HPLC, with each step repeated three times.

#### 2.5.5. In Vitro Release Analysis

Equal amounts of CUR-NCs^+^, CUR-NCs^−^, and CUR-CCs were dissolved in stimulated intestinal fluids (SIF), followed by dissolution analysis using a dissolution apparatus (RCZ-8A) (Tianjin Tianda Tianfa Technology Co., Ltd, Tianjing, China) [15]. The samples were subjected to agitation at 200 rpm and 37 °C. At intervals of 1, 2, 3, 5, 10, 15, 30, and 60 min, a 3 mL sample was withdrawn and centrifuged at 14,000 rpm and 4 °C for 15 min, and the supernatant was filtered through a 220 nm filter membrane. The concentration was determined using HPLC, with each step repeated three times.

#### 2.5.6. Stability

CUR-NCs with high and low concentrations were prepared (2 mg/mL, 0.5 mg/mL) and stored in sealed containers at 4 °C and 25 °C for 2 h, 4 h, 8 h, 24 h, and 7 days. The size and PDI were measured [29].

The same concentration of CUR-NCs^+^, CUR-NCs^−^, and CUR-NCs@Lipo-CS (0.5 mg/mL) were added to stimulated gastric fluids (SGF) and SIF at 37 °C, and the size and PDI were measured after 0.5 h, 1 h, 2 h, and 12 h.

### 2.6. Bioavailability in SD Rats

Male Sprague-Dawley (SD) rats (200–220 g) were provided by SPF (Beijing) Biotechnology Co., Ltd. (Beijing, China). All animal experiments were performed following the approved guidelines of the use and care of animals established by Jilin University.

Twenty-four male adult rats were fasted for 12 h but did not abstain from taking water. They were then randomly divided into four groups (n = 6) and administered CUR-CCs, CUR-NCs^+^, CUR-NCs@Lipo-CS, and CUR-NCs^−^, respectively. The dosage administered was 200 mg/kg, and 0.3 mL of blood was collected from the orbit at 0.166, 0.333, 0.5, 1, 2, 4, 6, 8, 10, and 12 h post-administration into anticoagulant Eppendorf [30]. The blood samples were centrifuged at 14,000 rpm and 4 °C for 15 min. The supernatant was then mixed with twice the amount of methanol to precipitate proteins, vortexed for 3 min, and centrifuged again. The resulting supernatant was transferred to Eppendorf, freeze-dried, reconstituted with methanol, and analyzed using the HPLC method to determine drug content. The measurement conditions were consistent with the previous analysis. Pharmacokinetic data were processed using DAS2.0 software [31].

### 2.7. Multiple-Particle Tracking In Vitro

Coumarin 6-labeled CUR-NCs^+^, CUR-NCs^−^, and CUR-NCs@Lipo-CS were diluted 10 times and dispersed in 0.1% mucin solution for 5 min. We added 10 μL of the dispersion to 200 μL of 5% mucus and put 20 μL on a glass slide and covered it. Slides should be sealed with mounting fluid to avoid dehydration and convection. Each sample was imaged at various positions simultaneously for 20 s [32,33]. Following enhancement with contrast and sharpening using Photoshop, diffusion analysis was performed using the ParticleTracker plugin in ImageJ (2.14.0) [34]. The MSD and effective diffusivities (*D*_eff_) were calculated using the following equations:

(2) $${MSD}_{\tau }={\left(x_{\left(t+\tau \right)}-x_{t}\right)}^{2}+{\left(y_{\left(t+\tau \right)}-y_{t}\right)}^{2},$$

(3) $$D_{\mathrm{eff}\;}=\frac{{MSD}_{\tau }}{4\tau },$$

where *x* and *y* represented the coordinates of the NPs, and *τ* is the time scale.

### 2.8. Cellular Uptake and Mechanisms

The conventional Caco-2 cell model was established, and CUR-NCs^+^, CUR-NCs@Lipo-CS, and CUR-NCs^−^ were introduced into the medium to reach a drug concentration of 100 μg/mL. Subsequent to drug administration, uptake was halted at 5 min, 10 min, 30 min, 1 h, and 2 h. After double PBS rinses, lysate was added, and samples were collected. Curcumin content per unit protein was quantified through the bicinchoninic acid (BCA) assay and HPLC.

Given that chitosan oligosaccharide is predominantly adsorbed onto liposomes, we have prepared coumarin 6-labeled blanks of liposomes (Lipo-C6), with half of these modified with chitosan oligosaccharide (Lipo-C6-CS) and half left untreated. Lipo-C6-CS or Lipo-C6 was added to the culture medium of Caco-2/E12 cells (3:7). After 1 h, the medium was collected, and the cells were rinsed three times with PBS and detached using trypsin. The fluorescence intensity of the cells was measured using a BD LSR Fortessa flow cytometer. The fluorescence intensity of the medium collected, and the initial medium was assessed using Infinite 200 PRO (Tecan, Männedorf, Switzerland), with excitation at 450 nm and emission at 505 nm. The mucoadhesive properties were calculated according to Equation (4):

(4) $$Mucoadhesive\;properties\;\left(\%\right)=100-\frac{OD\left(end\right)-OD\left(blank\right)}{OD\left(original\right)-OD\left(blank\right)}\times 100,$$

where *OD*(*end*) is the fluorescence intensity of the experimental groups, adding Lipo-C6-CS or Lipo-C6; *OD*(*original*) is the fluorescence intensity of the original medium with Lipo-C6-CS or Lipo-C6; and *OD*(*blank*) is the fluorescence intensity of pure medium.

For a deeper exploration of nanocrystal uptake mechanisms, Caco-2 cells were initially exposed to indomethacin, colchicine, quercetin, chlorpromazine, or an ice water bath. Subsequently, CUR-NCs^+^, CUR-NCs^−^, or CUR-NCs@Lipo-CS were introduced into the medium. Following a 1 h cellular uptake period, the curcumin content per unit protein was quantified using the BCA assay and HPLC.

### 2.9. Cell Cytotoxicity

The cytotoxicity of Caco-2 cells was assessed using the Cell Counting Kit-8 Assay. Cells were plated in 96-well plates at a density of 5 × 10^3^ cells per well and cultured for 24 h. Following medium removal, cells were exposed to CUR-NCs^+^, CUR-NCs^−^, or CUR-NCs@Lipo-CS at concentrations of 0.1, 0.5, 1.0, 2.0, 5.0, 10, and 20 μg/mL for 24 h. Subsequently, cells were washed twice with PBS and incubated in 10% CCK-8 solution, shielded from light for 90 min. The absorbance was read at 450 nm using Infinite 200 PRO (Tecan, Switzerland). Cell viability was determined using Equation (5):

(5) $$cell\;viability\left(\%\right)=\frac{OD\left(experiment\right)-OD\left(blank\right)}{OD\left(control\right)-OD\left(blank\right)}\times 100,$$

where *OD*(*experiment*) is the absorbance of the experimental groups, adding different forms of curcumin; *OD*(*control*) is the absorbance of the drug-free group containing only cells and medium; and *OD*(*blank*) is the absorbance of pure medium.

Similarly, the impact of inhibitors, CUR-NCs^+^, CUR-NCs^−^, and CUR-NCs@Lipo-CS, on cell viability at the designated experimental concentrations and time intervals was also examined.

### 2.10. Mucus Penetration

#### 2.10.1. Fluorescent Labeling of Nanocrystals

To create FRET pairs, 0.1 mg each of DiI and DiO were combined with 20 mL of DMSO along with CUR. The CUR-NCs^+^-FRET, CUR-NCs^−^-FRET, and CUR-NCs-FRET@Lipo-C6-CS (liposomes labeled with coumarin-6) were synthesized following established protocols.

Furthermore, to establish the association between CUR-NCs-FRET and the FRET phenomenon, approximately 0.5 mL of CUR-NCs-FRET was introduced into 5.0 mL of either pure water or water containing 60% methanol. Subsequently, the emission spectra of the samples were promptly recorded using an RF-6000 Spectrofluorometer at an excitation wavelength of 458 nm.

#### 2.10.2. 3D Mucus Penetration

To assess the interactions between CUR-NCs^+^, CUR-NCs^−^, and CUR-NCs@Lipo-CS with the mucus layer, Caco-2/E12 (7:3) cells were cultured on Transwell filter inserts (Corning) for 14–17 d. The medium was replaced by HBSS 30 min later, followed by replacing the upper HBSS with diluted CUR-NCs^+^-FRET, CUR-NCs^−^-FRET, and CUR-NCs-FRET@Lipo-C6-CS in the medium. The monolayers were then washed three times with HBSS, and the cells were stained with Hoechst 33342 (10 μg/mL) for 15 min at 37 °C. The cell layers supporting the membranes of the Transwell inserts were cut from the plastic support without fixation, mounted onto microscope slides, and covered with coverslips. The slides were immediately observed under a confocal microscope (LSCM, AXR(Ti2-E); Nikon, Tokyo, Japan). The sections were photographed using a laser confocal microscope in z-stack mode, and the results were reconstructed in 3D using NIS-Elements Viewer (5.21) software.

## 3. Results and Discussion

### 3.1. CUR-NCs Formulation Development and Optimization

#### 3.1.1. Preparation Methods for Screening CUR-NCs

**Figure 1:**
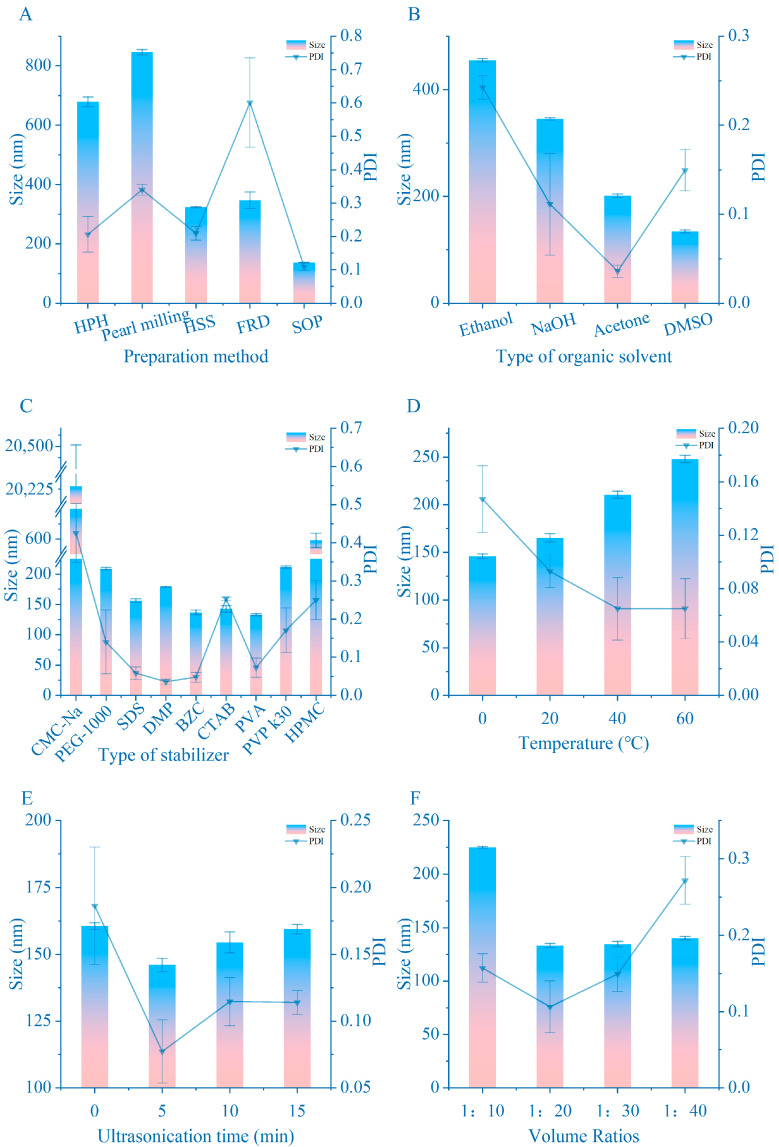
The effect of various parameters on size and PDI during nanocrystal preparation: (**A**) preparation method; (**B**) type of organic solvent; (**C**) type of stabilizer; (**D**) temperature; (**E**) ultrasonication time; (**F**) ratio of two phases. Data are means ± standard error of mean.

Numerous mainstream preparation methods exist for NCs. Figure 1A illustrates that NCs prepared via SOP exhibit significantly superior size and PDI compared to other groups. Specifically, the size range of NCs obtained through grinding is constrained by experimental conditions, leading to poor stability and susceptibility to agglomeration.

With the exception of FRD, the other four methods primarily utilize high shear forces from various sources to fragment crystals and reduce particle size. However, the limited shear force of the instruments in each method results in a theoretical minimum particle size. While repeated crushing may approach this limit and reduce PDI, it cannot further reduce particle size. Thus, we conclude that when the size of the NCs reaches the equipment’s limit, prescription becomes the primary determinant of particle size.

In principle, the FRD inhibits NCs’ aggregation and growth. As is known, in liquid dispersions, small particles dissolve more easily due to their larger specific surface area, resulting in increased drug concentration around them. This disrupts the dissolution balance, leading to continued drug crystallization and growth, with large particles as nuclei. Consequently, a smaller PDI indicates greater system stability. The FRD addresses this issue at its source, transitioning the system from an unstable liquid state to a stable solid state. However, this method has significant drawbacks: excess surfactant removal is impractical, and the product size becomes fixed upon NCs formation, allowing for size enhancement only through prescription optimization.

The microjet homogenizer utilized in this experiment lacks temperature control. Consequently, the high temperatures generated during operation cause NCs initially prepared via SOP to continue growing during homogenization, resulting in an increased NC size from 136.1 nm to 676.5 nm. The utilization of different instruments significantly contributes to the challenge of replicating the findings reported in the literature. While theoretically, higher shear forces in grinding and high-speed shearing yield smaller particles, in practice, nanoparticles smaller than 200 nm are challenging to achieve. The SOP, utilizing cavitation effects generated by ultrasound, applies large and uniform shear forces on CUR crystals, resulting in extremely uniform dispersion of prepared the NCs. Hence, this study ultimately selects the SOP.

#### 3.1.2. The Selection of the Organic Phase

Given our selection of the SOP method, the alternative organic solvent must exhibit a high diffusion coefficient in water to ensure rapid and uniform saturation of the drug in the solution, facilitating crystal precipitation [35]. As depicted in Figure 1B, crystals prepared using DMSO outperform those from acetone, ethanol, and sodium hydroxide solution groups in terms of size and PDI. Additionally, our experiments revealed that the acetone group yielded clusters after 24 h at 4 °C, a detrimental phenomenon. Furthermore, both the ethanol and NaOH groups exhibited varying degrees of agglomeration under the same conditions, whereas the DMSO group remained free from such issues. Moreover, given CUR’s enhanced solubility in DMSO, the reliance on organic solvents in product formulation can be further reduced. Consequently, DMSO was chosen as the organic phase for NCs preparation.

#### 3.1.3. Surfactant Selection

The choice of stabilizer not only determines the zeta potential of the final product but also dictates the complexity of subsequent formulation optimization and sets the lower limit for the size and PDI of the end product [36]. As depicted in Figure 1C, the SDS, PVP, PVA, CTAB, and BZC groups all demonstrated satisfactory performance. However, stability assessments revealed inadequacies in the SDS and CTAB groups, while the PVA group exhibited a smaller size compared to the PVP group. Consequently, BZC (a cationic surfactant) and PVA (an anionic surfactant) are earmarked for further use.

#### 3.1.4. Surfactant Concentration

**Figure 2:**
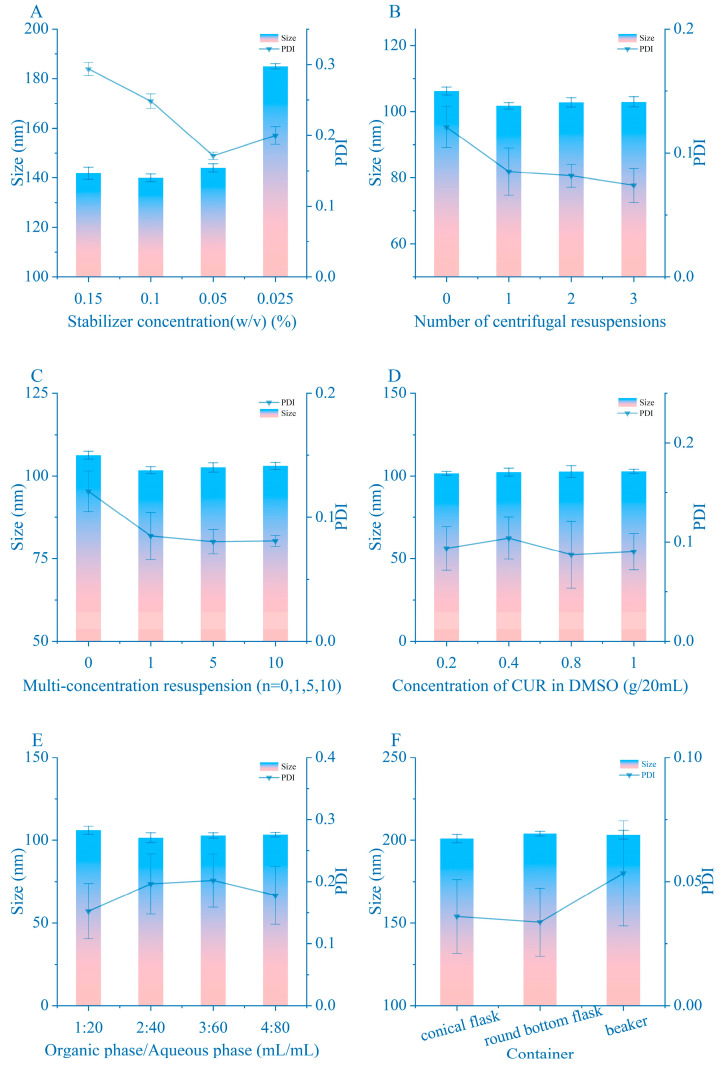
The effect of various parameters on size and PDI during nanocrystal preparation: (**A**) stabilizer concentration; (**B**) effect of multiple centrifugal resuspensions on particle size; (**C**) effect of centrifugal concentration on particle size; (**D**) effect of the concentration of the drug in the organic phase on the particle size; (**E**) effect of dose on particle size; (**F**) effect of experimental vessels on particle size. Representative images are presented. Data are means ± standard error of mean.

As illustrated in Figure 2A, increasing the surfactant concentration correlates with a reduction in product size; however, further escalation of surfactant concentration leads to an increase in PDI. This phenomenon arises from the dynamics of surfactant concentration relative to its critical micelle concentration (CMC). Below the CMC, the surfactant acts favorably by covering the NCs, thereby masking their hydrophobic layer—a beneficial effect. Conversely, exceeding the CMC results in the formation of free stabilizer micelles, contributing to the elevation of PDI [37]. Consequently, it is inferred that the optimal surfactant dosage is 0.05% (*w*/*v*).

In the preparation of the NCs, the most critical environmental factors are temperature, water-to-organic phase ratio, and ultrasonic time. The results are presented in Figure 1D–F. Notably, low temperature exhibits minimal impact on product size, underscoring why the size of prepared NCs increase following processing by a micro-jet homogenizer. A higher water phase ratio tends to reduce the size of CUR-NCs; however, indiscriminate escalation of this ratio does not consistently yield size reduction. The result obtained at a 1:20 ratio already represents the limit of NC size influenced by this factor. The presence and duration of ultrasound significantly affect NC size, but excessively prolonged ultrasound exposure should be avoided. The moment of crystallization is of significant importance with regard to the size of the nanocrystals. Ultrasound is an effective method of accelerating the homogeneous mixing of the organic phase with the mobile phase. Ultrasound induces bubble vibration and shear force, albeit with minimal impact on particle size and PDI in short durations due to energy dissipation. Consequently, the optimal conditions for NCs preparation are a water-to-organic phase ratio of 1:20 (*v*/*v*), a temperature of 4 °C, and ultrasonication for 5 min.

**Figure 3:**
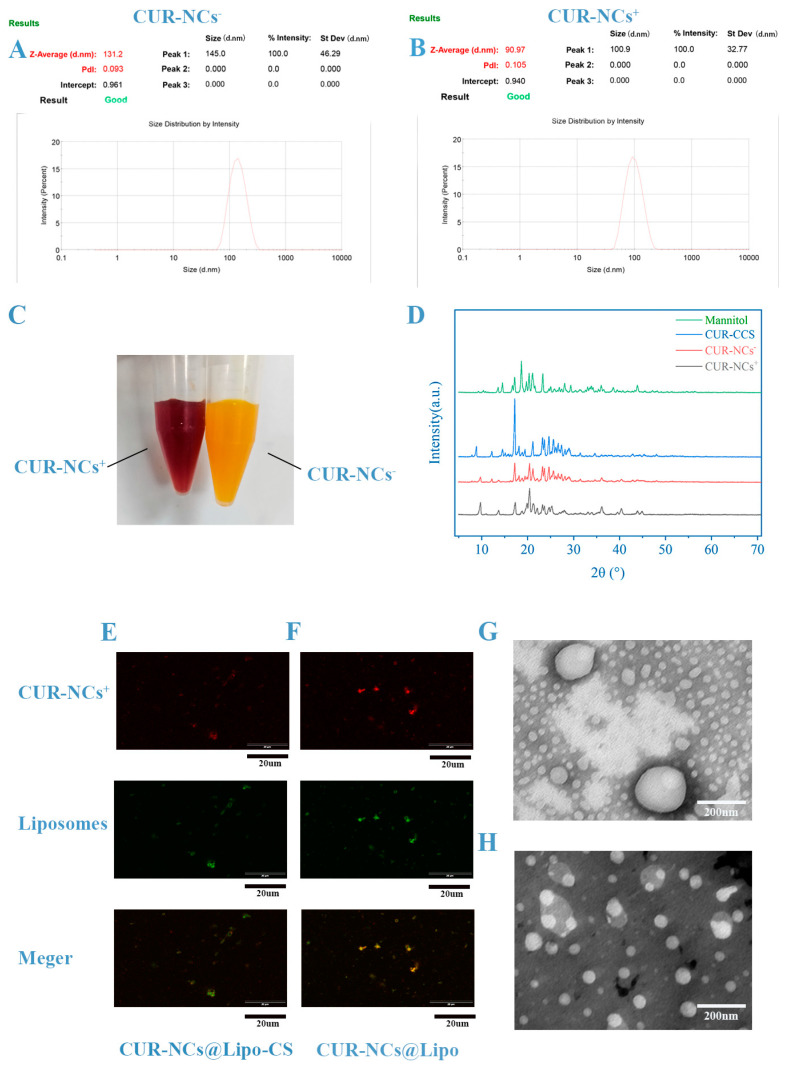
(**A**,**B**) Size and PDI of CUR-NCs^+^ and CUR-NCs^−^, measured via Malvern Nano-ZS. (**C**) Appearance of CUR-NCs^+^ and CUR-NCs^−^. (**D**) PXRD. (**E**,**F**) Laser scanning confocal fluorescence microscopy images of CUR-NCs@Lipo-CS and CUR-NCs@Lipo dissolved by water (scale bar is 20 μm). (**G**,**H**) TEM of CUR-NCs@Lipo and CUR-NCs@Lipo-CS. The number of liposomes represented in the images is indicative of the actual degree of encapsulation (concentrations of CUR-NCs@Lipo and CUR-NCs@Lipo-CS are same; scale bar is 200 nm). Representative images are presented.

Minimizing the quantity of materials in the drug preparation process enhances its economic, safety, and environmental attributes. As previously concluded, a higher proportion of the water phase results in NCs approaching their size limit; however, this corresponds to reduced drug concentration, increased surfactant usage, and greater water volume for removal, presenting a drawback. To counter this, resuspending samples with the supernatant removed during centrifugal resuspension increases drug concentration while eliminating free active agents and residual organic solvents. Interestingly, multiple resuspension cycles have no effect on the final product’s size and PDI, while CUR-NCs may be slightly affected by dissolved content in the solution, resulting in minor size reduction upon supernatant removal (Figure 2B,C). Moreover, factors such as drug concentration, amplification experiments, and sample preparation containers (Figure 2D–F) exhibit no significant impact on the final product’s size and PDI. Nevertheless, if the conditions governing the prescription of the aforementioned factors are not optimized, the aforementioned every single factor may affect the experimental results. For instance, when CTAB was employed as a stabilizer in our experiments, the samples prepared were only able to yield nanocrystals of a small size if a conical flask was utilized and 20 mL of sample was prepared at a time. In addition, by observing the color of the sample, it is also easy to determine the size of the sample; the closer the color of the sample is to reddish brown (Figure 3C), the smaller the sample is; if the size of the sample is greater than 100, the color is closer to yellow (Figure 3C). After analyzing different nanocrystal preparation conditions, it was determined that the optimization challenge varies across parameters, impacting size and PDI in the following order: surfactant type > organic solvent type > ratio of two phases > temperature > surfactant dosage > ultrasonication time > others. 

In summary, the optimal formulation for NCs is as follows: dissolve 1.0 g of CUR in 20 mL of DMSO, add 2 mL of this solution to 40 mL water with 0.05% (*w*/*v*) PVA or BZC, and ultrasonicate it at 4 °C for 5 min. This results in the final product with a size of 131.2 nm and a PDI of 0.093 for the PVA group, with a zeta potential of −4.52 mV, and a product with a size of 90.97 nm and a PDI of 0.105 for the BZC group, with a zeta potential of +54.6 mV.

### 3.2. PXRD

As shown in Figure 3C, the PXRD results show that CUR has characteristic absorption peaks at 17.14°, 20.52°, 21.24°, 23.18°, 24.56°, and 28.10°.The same characteristic peaks can be observed in the PXRD spectrum of CUR-NCs^+^ and CUR-NCs^−^. Therefore, it is believed that the crystal form of CUR did not change during the preparation of NCs.

### 3.3. TEM

**Figure 4:**
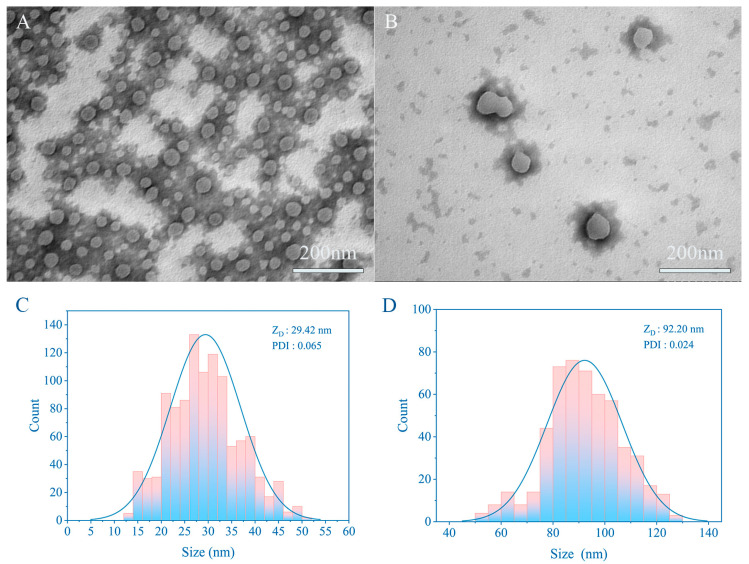
TEM images of CUR-NCs^+^, CUR-NCs^−^, and particle size distribution: (**A**,**C**) CUR-NCs^+^; (**B**,**D**) CUR-NCs^−^ (concentration of CUR-NCs^+^ and CUR-NCs^−^ are the same; scale bar is 200 nm). Representative images are presented.

Laser particle size analyzers utilize light scattering (diffraction) by particles to determine particle size. Essentially, when light encounters particles during propagation, some of it deviates from its original direction. The degree of deviation increases with smaller particle sizes and decreases with larger ones. While this method efficiently and straightforwardly measures particle size, errors become prominent when the size is <100 nm. Therefore, the precise particle size requires confirmation via scanning electron microscopy or transmission electron microscopy. The presence of a hydrated layer also results in larger measurements from the particle sizer than those observed in the electron micrographs. Figure 4 illustrates that the size of CUR-NCs^+^ prepared according to the prescribed method is only 29.42 nm. Additionally, CUR-NCs^−^, with a size of 92.20 nm, also exhibit commendable nano-properties.

### 3.4. Fabrication of Liposomes

The exceptional dissolution characteristics of nanocrystals have propelled their extensive integration into the development of BCS class-II drug models. However, BCS-IV drugs exhibit poor biomembrane permeability and susceptibility to P-gp. If nanocrystals can be efficiently internalized by cells while leveraging their potent drug-carrying capabilities, a substantial enhancement in the bioavailability of BCS class IV drugs is anticipated. Consequently, swift dissolution of nanocrystals is not envisaged during oral absorption. Encapsulation within liposomes serves a dual purpose: shielding nanocrystals from aggregation and expansion induced by diverse external factors like digestive fluids and mucous layers, while concurrently mitigating premature dissolution and enhancing overall bioavailability. The incorporation of E-TPGS into liposomes has demonstrated an inhibitory effect on P-gp, thereby further enhancing bioavailability.

Regarding the question of whether nanocrystals are encapsulated by liposomes, we disagree with the use of fluorescence colocalization, which often leads to false conclusions. In Figure 3E,F, we can clearly see that there is more fluorescence overlap in CUR-NCs@Lipo compared to CUR-NCs@Lipo-CS, which does not mean that the incorporation of oligochitosan ruptures the liposomes. Interestingly, during the preparation of CUR-NCs@Lipo, we found that CUR-NCs@Lipo-CS was positively charged and had a particle size of around 170 nm, whereas CUR-NCs@Lipo was negatively charged and had a particle size of around 200 nm. The addition of oligochitosan makes the particle size of the nanoparticles smaller, and there seems to be a high probability that the addition of oligochitosan caused this result; however, through the electron microscope image of CUR-NCs@Lipo (Figure 3G,H), we found that due to the interactions between the charges, the liposomes adsorbed a ring of nanocrystals at the periphery, resulting in the large size of the measured particle, whereas the addition of chitosan reversed the charge of the liposome, turning the attraction into mutual repulsion, and the liposome not wrapped around the nanocrystals shows its original size. Then, we found another interesting phenomenon from the electron micrographs, the encapsulation rate of liposomes increased after the addition of chitosan, and the number of liposomes encapsulated with nanocrystals increased significantly. This may be due to the fact that the inside of the liposome is still negatively charged, and the outside environment is positively charged, and this potential difference drives the nanocrystals to enter the liposome, resulting in a significant increase in the encapsulation rate.

### 3.5. In Vitro Release

**Figure 5:**
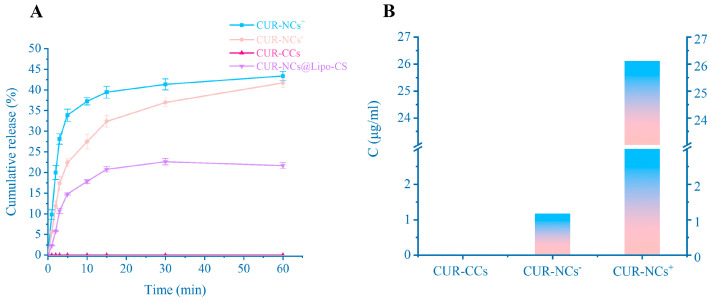

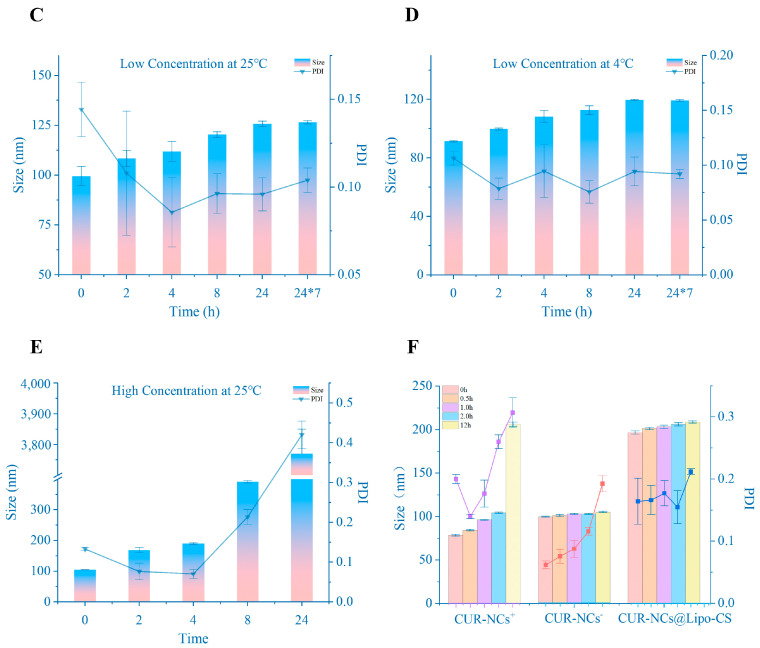
(**A**) Dissolution profile of CUR-NCs@Lipo-CS, CUR-NCs^+^, CUR-NCs^−^ and CUR-CCs (n = 3). (**B**) Saturation solubility of CUR-NCs^+^ and CUR-NCs^−^ (n = 3). (**C**) Stability of low-concentration nanocrystals at 25 °C (n = 3). (**D**) Stability of low-concentration nanocrystals at 4 °C (n = 3). (**E**) Stability of high-concentration nanocrystals at 25 °C (n = 3). (**F**) Stability of CUR-NCs^+^, CUR-NCs^−^ and CUR-NCs@Lipo-CS in Sif (n = 3). Representative images are presented. Data are means ± standard error of mean.

Figure 5A shows the cumulative release curves of CUR-NCs^+^, CUR-NCs^−^, CUR-NCs@Lipo-CS, and CUR-CCs. It can be seen that CUR-CCs is insoluble in water and cannot be measured in water. Regardless of whether it is cationic or anionic, NCs can detect a large amount of CUR in the solution, but since the size of NCs^+^ is smaller than that of NCs^−^, according to the Noyes–Whitney equation:

(6) $$\frac{dC}{dt}=kA\left(C_{s}-C_{t}\right),$$

where *dC*/*dt* is the dissolution rate, *A* is the surface area, *k* is a constant, *C_s_* is the saturated solubility of the drug, and *C_t_* is the drug concentration [38,39].

It is not difficult to find that the larger the specific surface area, the greater the dissolution rate of the drug, so CUR-NCs^+^ has a higher release rate than CUR-NCs-. In addition, different surfactants have different solubilizing effects on drugs, making CUR-NCs^+^ exhibit higher saturated solubility. At the same time, considering the influence of charge, we also prepared CUR-NCs without adding any surfactant. There is no doubt that this group of CUR-NCs has extremely poor stability and rapid polymerization. PDI > 0.7 and Zeta = −16.7 mV imply that CUR is naturally attracted to cationic surfactants, resulting in a better performance of CUR-NCs^+^ than CUR-NCs^−^ at the physical level, but we did not conduct similar experiments with other drugs to corroborate this idea. The slow and limited dissolution of CUR-NCs@Lipo-CS is a consequence of the protective effect of the liposome membrane on the nanocrystals. Since the size of the CUR-NCs is much smaller than the 220 nm pore size of the filter membrane, in order to increase the accuracy of the content determination, we removed the CUR-NCs suspended in the solution via centrifugation before the content measurement. We considered the measured concentration to be the true concentration of saturated solubility about CUR-NCs.

### 3.6. Stability Studies

Figure 5C is the change of size of CUR-NCs^+^ at low concentration and room temperature, Figure 5D is the change of size of CUR-NCs^+^ at low concentration and low temperature, and Figure 5E is the change of size of high-concentration CUR-NCs^+^ at room temperature, as the time go by. Comparing C, D, and E, it can be seen that CUR-NCs^+^ are sensitive to heat. The storage size of CUR-NCs^+^ at room temperature was larger than that at low temperature. This phenomenon is more obvious in the high-concentration group. At the same time, we observed that when CUR-NCs^+^ were still at the nanoscale, although the size increased slightly with time, it eventually stabilized, and the PDI continued to become smaller with time. It is not difficult to understand that small NCs dissolve and precipitate on large crystals, causing the size of the crystals to concentrate gradually; thus, the PDI decreases, and the system reaches a more stable state [40]. At the same time, due to the increase in concentration, the number of particles in the system increases and the entropy value increases, which means that the system is more unstable. In addition, the CUR-NCs in the high-concentration group are at room temperature. The increase in temperature further increases the entropy value, which leads to the CUR-NCs continuing to grow and aggregate, eventually losing their nanometer properties. After a week, all the crystals aggregate and sink to the bottom of the container. In general, although the high-concentration group is not as stable as the low-concentration group, the overall stability is still excellent during low-temperature storage, and the size change is minimal within a week. Therefore, it is believed that the stability of CUR-NCs prepared under this prescription condition is excellent.

The most apparent change in particle size is observed in CUR-NCs^+^, while the change in particle size of CUR-NCs^−^ is less pronounced. However, the PDI of CUR-NCs^−^ has increased significantly and is still on an upward trajectory. In contrast, CUR-NCs@Lipo-CS exhibits minimal changes in both particle size and PDI, suggesting that CUR-NCs^+^, which is rarely influenced by the enzyme and electrolytes in SIF under the protection of liposome, does not undergo aggregation.

### 3.7. Bioavailability

**Figure 6:**
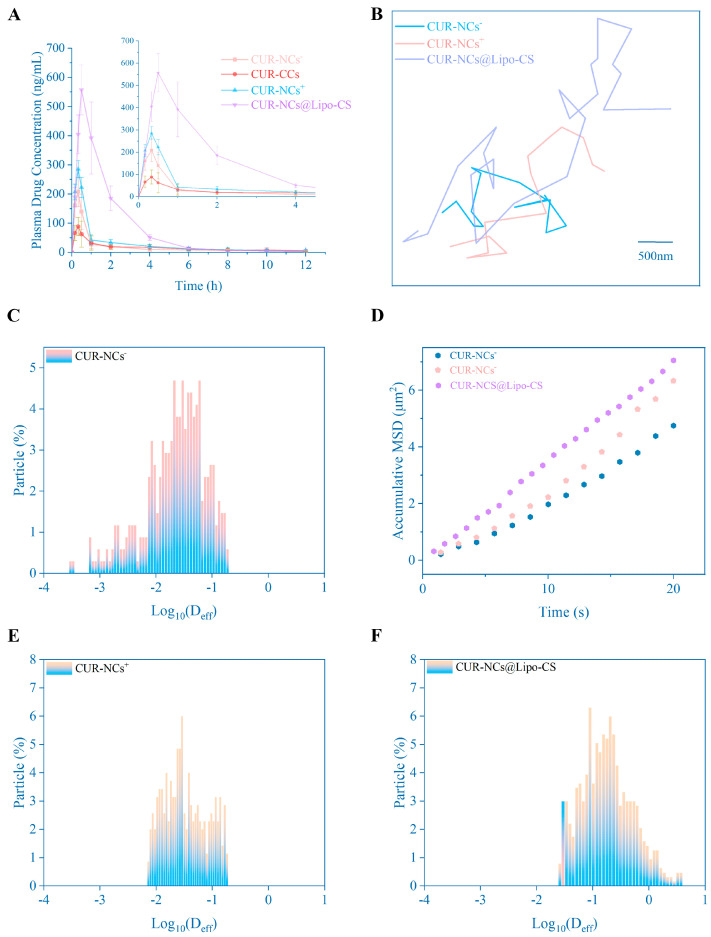
(**A**) Plasma concentration–time curves of oral CUR-NCs^+^, CUR-NCs^−^, CUR-CCs, and CUR-NCs@Lipo-CS (n = 6). (**B**) Mobility of CUR-NCs^+^, CUR-NCs^−^, and CUR-NCs@Lipo-CS at 37 °C. Representative trajectories of particle motion in 20 s. Scale bar is 500 nm. (**D**) MSD values as a function of time. (**C**,**E**,**F**) Distribution of the logarithms of individual particle effective diffusivities (*D*_eff_) on a timescale of 20 s. Representative images are presented.

Using SD rats as the animal model, Figure 6A presents the drug–time curve after oral administration of each preparation. As shown in the results in Table 2, the C_max_ values of CUR-NCs@Lipo-CS, CUR-NCs^+^, and CUR-NCs^−^ are 6.006, 3.098, and 2.251 times that of CUR-CCs. The AUG _(0–∞)_ values of CUR-NCs@Lipo-CS, CUR-NCs^+^, and CUR-NCs^−^ are 5.427, 1.596. and 1.22 times that of CUR-CCs. These data all reflect that the nanocrystal delivery method has greatly improved the bioavailability of curcumin, among which CUR-NCs@Lipo-CS has the best effect.

### 3.8. Multiple-Particle Tracking

The trajectory of CUR-NCs@Lipo-CS, CUR-NCs^+^, and CUR-NCs^−^ within the mucus layer is illustrated in Figure 6B. Notably, within the same scale, CUR-NCs@Lipo-CS exhibit a broader motion range, indicative of Brownian motion, suggesting superior mucus penetration compared to CUR-NCs^+^ and CUR-NCs^−^ [32,33]. Mean square displacement (MSD) was calculated on a 20 s time scale (Figure 6D), revealing that, on average, the MSD of CUR-NCs@Lipo-CS was approximately 1.46 times greater than that of CUR-NCs^−^. Additionally, the effective diffusivity (D_eff_) distribution shown in Figure 6C,E,F also shows that CUR-NCs@Lipo-CS have higher diffusivity. Thus, CUR-NCs@Lipo-CS are more adept at traversing the mucus barrier and accessing the upper cells of the small intestinal mucosa, consequently yielding enhanced bioavailability.

### 3.9. Cellular Uptake of CUR

#### 3.9.1. Determination of Cellular Uptake of CUR

**Figure 7:**
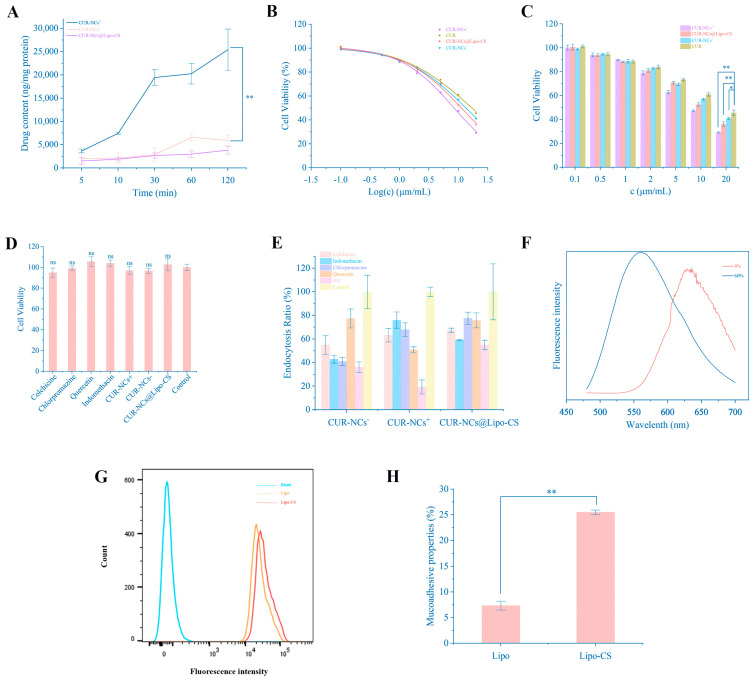

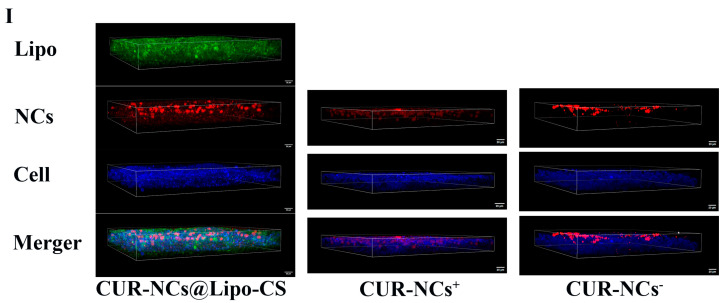
(**A**) Cellular uptake of CUR formulation on Caco-2 cells (n = 3 per group). (**B**) The IC50 value of Cur-LPs, Cur, and Cur-NCs in macrophages. (**C**) In vitro evaluation of Caco-2 cells’ viability treated with CUR-NCs@Lipo-CS, Cur, Cur-NCs^−^, and Cur-NCs^+^ after 24 h (n = 3 per group). (**D**) In vitro cytotoxicity of endocytic inhibitors and CUR formulation based on Caco-2 cells (n = 3 per group). (**E**) Possible uptake mechanisms investigated via cellular uptake of CUR formulation (n = 3 per group). (**F**) Fluorescence emission spectra of ETO-NCs-FRET dissolved by water or methanol. (**G**) Flow cytometry analysis of cellular uptake and mean fluorescence intensities of Caco-2 cells incubated with coumarin 6-labeled Lipo-C6-CS and Lipo-C6. (**H**) Mucoadhesive properties of Lipo and Lipo-CS (n = 3 per group). (**I**) Uptake by mucus-containing E12/Caco-2 cells that simulated the multiple barriers of intestinal mucosa (scale bar is 20 μm). The proximity of the red component to the bottom indicates a heightened resistance of the nanocrystals to the cellular barrier. Representative images are presented. Data are means ± standard error of mean. ns, no significant difference; * *p* < 0.05 and ** *p* < 0.01 via one-way ANOVA and Bonferroni’s test.

The utilization of Caco-2 as a model to mimic the uptake of CUR-NCs@Lipo-CS, CUR-NCs^+^, and CUR-NCs^−^ indicated a notably higher cellular uptake of CUR-NCs^+^ at all time points compared to CUR-NCs@Lipo-CS and CUR-NCs^−^ (Figure 7A). These results underscore the pivotal role of particle size in influencing nanocrystal uptake. The relatively diminished cellular uptake of CUR-NCs@Lipo-CS can be ascribed to the inherent constraints of the liposome membrane in releasing encapsulated CUR-NCs. Over time, the cellular uptake of CUR-NCs is modulated by P-gp, with transporter protein availability and energy determining the gradual accumulation of curcumin to an equilibrium state. In contrast, due to distinct transmembrane transport mechanisms and enhanced equilibrium solubility, CUR-NCs^+^ with a smaller particle size did not reach equilibrium within 120 min. Furthermore, intracellular curcumin accumulation continued to rise gradually, elucidating the rationale behind the lowest observed IC50 value (Figure 7B,C). Despite the consistently lower cellular uptake of CUR-NCs@Lipo-CS compared to CUR-NCs, intracellular curcumin content increased steadily. This phenomenon contributes to the explanation for the smaller IC50 value of CUR-NCs@Lipo-CS.

#### 3.9.2. Mechanisms of Cellular Uptake of CUR

Chlorpromazine serves as an inhibitor of clathrin-mediated endocytosis, while indomethacin inhibits caveolae-mediated endocytosis. Colchicine acts as an inhibitor of macropinocytosis, and quercetin inhibits clathrin/caveolae-independent endocytosis [41]. A temperature of 4 °C primarily slows down cellular energy metabolism, thus impeding active transport. As depicted in Figure 7, the uptake of CUR-NCs@Lipo-CS, CUR-NCs^+^, and CUR-NCs^−^ is an energy-intensive process. Moreover, the data (Figure 7E) indicate that various inhibitors exert inhibitory effects on cellular uptake, suggesting the involvement of multiple uptake pathways. Non-grid- and non-nubbin-mediated endocytosis is the primary internalization mechanism for CUR-NCs^+^, whereas nubbin-mediated endocytosis is the likely route for CUR-NCs@Lipo-CS. Cell viability experiments revealed that the addition of inhibitors, including CUR-NCs@Lipo-CS, CUR-NCs^+^, and CUR-NCs^−^, did not significantly impact cell viability throughout the experiments.

#### 3.9.3. Fluorescence Properties

To determine if CUR-NCs are absorbed into cells as intact crystals with enhanced clarity, Dio and DiI were utilized together. The maximum emission wavelength shifted from 600 nm to 500 nm due to the transition of CUR-NCs-FRET from a colloidal to a solute state. This shift signifies the generation of the FRET signal solely within the CUR-NCs, vanishing upon crystal dissolution. Hence, the red fluorescence observed is emitted exclusively by the nanocrystals (Figure 7F).

#### 3.9.4. Mucoadhesive Properties of Polycationic Chitosan Oligosaccharide

Chitosan, renowned for its exceptional hydrophilicity and positive charge, interacts favorably with the negative charge of the mucus layer, significantly prolonging drug residence time in the gastrointestinal tract when employed as a drug delivery carrier. To assess if the chitosan oligosaccharide utilized could elicit comparable effects in enhancing adhesion, mucoadhesive properties, and uptake of Lipo-C6-CS and Lipo-C6, investigations were conducted. As illustrated in Figure 7G,H, chitosan oligosaccharide proved effective in augmenting cell adhesion to liposomes; however, the improvement in cellular uptake was constrained.

#### 3.9.5. Mucus Penetration on Caco-2/E12 Cells

Nonetheless, the existence of a mucus layer in the gastrointestinal tract hindered the accurate simulation of the authentic uptake by Caco-2 cells. Consequently, we opted to co-culture E12 cells with Caco-2 cells to replicate cellular uptake in the presence of the mucus layer. Figure 7I clearly demonstrates that CUR-NCs@Lipo-CS is more likely to penetrate the mucus layer and be taken up by cells than CUR-NCs^+^ and CUR-NCs^−^. Furthermore, the aggregation and deposition of CUR-NCs is reduced under the protection of liposomes. A considerable number of small particles traverse the cell layer. However, the cellular uptake of CUR-NCs^+^ is still greater than that of CUR-NCs^−^. Both CUR-NCs^+^ and CUR-NCs^−^ were primarily concentrated in the upper mucus layer of the cells and were predominantly aggregated, which is consistent with the previous experimental results.

## 4. Conclusions

In this study, the sonoprecipitation method was utilized to successfully synthesize cationic curcumin nanocrystal liposomes. The morphology of CUR-NCs@Lipo-CS was confirmed through a series of in vitro characterization experiments, validating the successful entrapment of nanocrystals within the lipid bilayer, enhanced by chitosan oligosaccharide. In vitro assays demonstrated a marked decrease in the rate and extent of CUR-NCs@Lipo-CS release from SIF in comparison to nanocrystals alone, highlighting the lipid bilayer’s efficacy in impeding rapid core nanocrystal dissolution. Cell uptake studies indicated that CUR-NCs@Lipo-CS displayed superior mucus-penetrating capabilities, effectively mitigating the mucosal barrier’s impact on nanocrystal absorption. These results emphasize the heightened potential for oral bioavailability of CUR-NCs@Lipo-CS in delivering BCS-IV drugs, affirming the success of pharmaceutical strategies in translating dissolution enhancement into comprehensive nanocrystal uptake within NCs-based oral BCS IV delivery systems.

Moreover, we recommend that our colleagues follow the sequence of screening the organic phase types, surfactant types, and ultrasound time and prepare nanocrystals of different drugs under low-temperature, high-water-ratio, and high-surfactant-concentration conditions in order to prepare the ideal nanocrystals.

## Figures and Tables

**Table 1:** Independent factors in the preparation of CUR-NCs.

Independent Variables	Level			
Temperature (°C)	0	20	40	60
Ultrasonic time (min)	0	5	10	15
Organic phase/aqueous phase (*v*/*v*)	1:10	1:20	1:40	1:100
Stabilizer concentration (% *w*/*v*)	0.15	0.10	0.05	0.025

**Table 2:** Pharmacokinetic parameters of oral CUR-NCs^+^, CUR-NCs^−^, CUR-CCs, and CUR-NCs@Lipo-CS.

Parameter	CUR-CCs (po)	CUR-NCs^+^ (po)	CUR-NCs^−^ (po)	CUR-NCs @Lipo-CS
T_max_ (h)	0.361 ± 0.068	0.361 ± 0.068	0.333	0.5
t_1/2_ (h)	3.182 ± 1.096	3.534 ± 1.467	5.796 ± 2.727	2.384 ± 0.44
C_max_ (μg/L)	92.785 ± 37.442	287.493 ± 26.511	208.871 ± 51.702	557.311 ± 85.443
AUC_(0–t)_ (μg·h/L)	189.899 ± 95.831	343.107 ± 81.295	233.062 ± 52.893	1030.536 ± 212.234
AUC_(0–∞)_ (μg/L·h)	209.477 ± 118.04	381.828 ± 99.082	305.729 ± 111.782	1047.894 ± 208.003

## Data Availability

Further inquiries can be directed to the corresponding authors.
